# The Correlation between Hemoglobin A1c (HbA1c) and Hyperreflective Dots (HRD) in Diabetic Patients

**DOI:** 10.3390/ijerph17093154

**Published:** 2020-05-01

**Authors:** Bing Seng Wong, Sharanjeet Sharanjeet-Kaur, Nor Fariza Ngah, Rajan Rajasudha Sawri

**Affiliations:** 1 Optometry and Vision Science Program, Faculty of Health Sciences, Universiti Kebangsaan Malaysia, Jalan Raja Muda Abdul Aziz, 50300 Kuala Lumpur, Malaysia; vincent777070@gmail.com; 2 Department of Ophthalmology, Hospital Shah Alam, Persiaran Kayangan, Seksyen 7, 40000 Shah Alam, Selangor, Malaysia; drfarizangah@gmail.com (N.F.N.); sudharajan81@gmail.com (R.R.S.)

**Keywords:** hyperreflective dots, HbA1c, diabetic retinopathy

## Abstract

Hyperreflective dots (HRD) are activated retinal microglial cells induced by retinal inflammation in diabetic patients. This study was conducted to compare the HRD count of normal and diabetic subjects; to determine the correlation between hemoglobin A1c (HbA1c) levels and HRD count; to determine HbA1c cut-off levels for the appearance of HRD in diabetic patients. A cross-sectional study was conducted among normal and diabetic patients. Fundus photos, SD-OCT images and HbA1c levels were taken. A total of 25 normal subjects, 32 diabetics without retinopathy and 26 mild-to-moderate nonproliferative diabetic retinopathy (NPDR) diabetics were recruited. There was a statistically significant difference between the mean count of HRD among the normal group, the diabetic without retinopathy group and the mild-to-moderate NPRD group. The mean HRD count in the inner retina layer was significantly higher compared to the outer retina layer. There was a significant linear relationship between the HbA1c levels and HRD count. Using the receiver operating curve, the HbA1c level of 5.4% was chosen as the cut-off point for the appearance of HRD. The positive linear correlation between the HbA1c levels and the appearance of HRD may indicate that hyperglycemia could activate retina microglial cells in diabetic patients.

## 1. Introduction

Diabetic retinopathy (DR) is a chronic progressive, potentially sight-threatening disease of the retinal microvasculature, and is associated with prolonged hyperglycemia and other conditions linked to diabetes mellitus such as hypertension [1], hyperlipidemia, dysregulated hormones levels and growth factors. These induce a cascade of biochemical and physiological changes that lead to neurovascular damage in the retina through oxidative stress, inflammation and apoptosis [2]. High blood sugar levels (hyperglycemia) in diabetes mellitus is considered the most causative etiology for DR [3]. A hyperglycemia environment in the retina can stimulate the accumulation of inflammatory mediators and reactive oxygen species (ROS), which induce the activation of microglia cells [4]. Other than these conditions, studies have suggested a strong relationship between elevated plasma homocysteine and DR [5,6,7,8]. However, a systematic review has shown that there is an association between elevated levels of plasma homocysteine and the risk of DR, but this association was stronger for type 1 DM (odds ratio = 1.83, 95% confidence interval, 1.28–2.62) than type 2 DM (odds ration = 1.59, 95% confidence interval, 1.28–2.62) [9].

In hyperglycemia, the activated microglia cells secrete pro-inflammatory molecules and cytokines used for phagocytosis and the destruction of damaged cells, which arise to retinal neurodegeneration [10]. Retinal neurodegeneration and inflammation are suggested as the early events that precede the clinical signs of DR in diabetic patients. Retinal microglia cell activation has been shown to cause initial inflammatory response, and is capable of causing vascular abnormalities and neuronal degeneration [3]. 

Hyper-reflective dots (HRD) are described as the activation of microglia cells (aggregation of microglial cells) [11]. HRD are often described as small, mostly punctiform, and highly reflective dots which can be counted quantitatively on SD-OCT B-scans, in both the inner retina (IR) and the outer retina (OR). They are believed to act as valuable SD-OCT findings for diagnosis, prediction of disease progression, and follow-ups in diabetic patients [10,11]. HRD have also been observed in other retinal diseases such as age-related macular degeneration (ARMD) and retinal vein occlusion (RVO) [12,13,14]. 

N-terminal valine residue of erythrocyte hemoglobin become irreversibly glycosylated in proportion to circulating glucose concentrations, and the resultant product is referred to as hemoglobin A1c (HbA1c) [15]. In comparison with fasting blood sugar (FBS), HbA1c can provide a long-term glycemic value, as the life span of glycosylated hemoglobin, which is approximately 120 days, is highly correlated with fasting blood sugar (FBS) measurements [16], and does not require measurement in the fasting state.

This study aims to compare the HRD count in normal subjects, diabetic subjects without retinopathy, and those with mild non-proliferative diabetic retinopathy (NPDR); to determine the correlation between HbA1c levels and HRD count; and to determine the HbA1c cut-off level for the appearance of HRD in diabetic patients.

## 2. Materials and Methods 

### 2.1. Sampling

This was a prospective, cross-sectional study with purposive sampling among normal and diabetic patients who attended an ophthalmology clinic in a government hospital in the Klang Valley, Selangor, from September to December 2018. The inclusion criteria for diabetic patients were type 2 diabetes without retinopathy, and mild-to-moderate NPDR; normal subjects were to show no signs of any retinal or choroidal diseases. The exclusion criteria were diabetic macular edema (DME), previous retinal treatment, previous intraocular surgery, myopia greater than −6 diopters, age below 18 years old, and pregnant women. 

This study was conducted according to the tenets of the Declaration of Helsinki of human subjects, and research approval was obtained from the Research Ethics Committee, Universiti Kebangsaan Malaysia (NN-2018-117) and the Medical Research and Ethics Committee (MREC) Ministry of Health Malaysia (NMRR-18-1101-40586). Written informed consent was obtained from the subjects after a detailed explanation of the study. 

### 2.2. Sample Size Calculation

**Figure 1:**
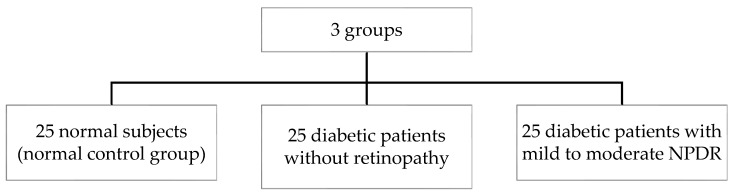
Grouping of subjects.

Power analysis for a one-way analysis of variance (ANOVA) with three groups was conducted in G*Power 3.1 to determine a sufficient sample size using an alpha of 0.05, a power of 0.80, and a large effect size (f = 0.40) [17]. Based on the software calculation, the desired sample size was 66. Taking into account the drop-out rate of 10%, a total of 75 subjects (eyes) divided into three groups as shown in Figure 1, were required for this study. Only one eye of each subject was used for the SD-OCT analysis. 

### 2.3. Procedure

Subjects who passed the diabetic screening (normal control group) or underwent ophthalmology examination (diabetic study group) by an ophthalmologist at the government hospital were selected to have fundus photos taken using a Canon CR-2 non-mydriatic camera, SD-OCT images scanned by a Zeiss Primus 200, and HbA1c levels determined by the high-performance liquid chromatography (HLPC) method. 

### 2.4. HRD Image Grading 

**Figure 2:**
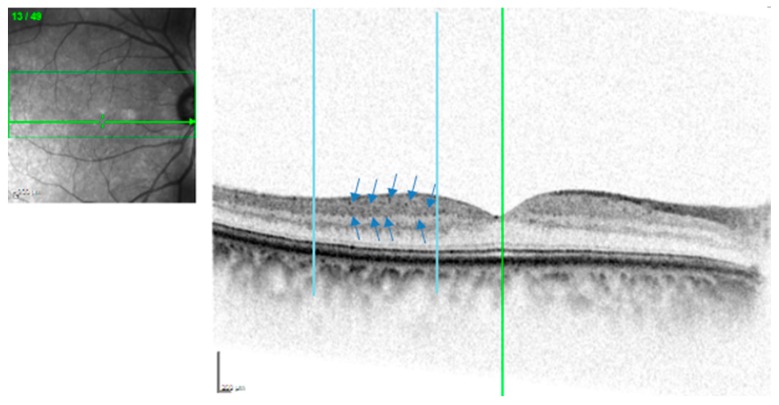
Spectral domain OCT linear scans in the macula. Two vertical lines were traced at 500 μm and 1500 μm from the center of the fovea in the temporal region. The blue arrow indicates the presence of Hyperreflective dots (HRD).

In search of the presence of HRD, single 1800 SD-OCT line scans (6 mm in length), which fixate on the center of the fovea, were analyzed for each patient. Two red vertical lines were traced at 500 μm and 1500 μm from the center of the fovea in the temporal region [5], thus excluding the foveal avascular zone, as shown in Figure 2. A manual count of the HRD, defined as small, punctiform, white lesions, was performed by two masked examiners. The location (inner or outer retina) and size of HRD were also recorded. The HRD, which were identified on the OCT B-scan were then compared with their corresponding image on the en face map and their color fundus photo, in order to exclude exudates and microaneurysms which mimic the hyperreflective appearance of HRD. Only one eye of each subject was used for the SD-OCT analysis. 

### 2.5. HbA1c Levels

The automated cation-exchange high performance liquid chromatography (HPLC) Bio-Rad D-10 Hemoglobin Testing System was used [18]. It utilizes principles of ion-exchange. The samples were automatically diluted and injected into the analytical cartridge. The D-10 delivered a programmed buffer gradient of increasing ionic strength to the cartridge. The hemoglobins were separated based on their ionic interactions with the cartridge material. The separated hemoglobins then passed through the flow cell of the filter photometer, where exchanges in the absorbance at 415 nm were measured. 

Blood samples were collected in a vacuum collection tube containing EDTA by nurses at the hospital. The samples were then sent to the lab for analysis. For the determination of HbA1c, a lysis of the blood cells was first performed. The samples were incubated at 37 °C to eliminate the unstable aldimine form. After centrifugation, the supernatant was injected into the HPLC system. The gradient separation via HPLC at 30 °C lasted 5 min. The chromatograms were recorded by a UV detector. The quantification was performed with the delivered blood calibrator, and the concentration was calculated via the integration of the peak heights. A sample report and a chromatogram were generated for each sample. 

### 2.6. Statistical Analyses

Statistical analyses for this study were performed using the Statistical Package for the Social Sciences (SPSS) version 22.0 for Windows (SPSS inc, Chicago, IL, USA). A p-value less than 0.05 was defined as statistically significant. The Kolmogorov–Smirnov test was used to test normality of the data distribution. A descriptive analysis was used to illustrate the mean value; standard deviation (SD); median; range and percentage of HRD numbers; HbA1c levels; age; gender; duration of diabetes.

Cohen’s kappa coefficient was used to measure intra and inter-rater agreements for the image grading of the HRD. Comparison in the number of HRD between the normal control group and diabetic groups was conducted by means of ANOVA. Pearson’s correlation coefficient was used to determine the correlation between the HbA1c levels and HRD count. A receiver operating characteristic (ROC) curve was plotted to determine the HbA1c cut-off level for the appearance of HRD, and the cut-off point of the number of HRD to detect diabetes.

## 3. Results

Although the calculation of the sample size required a minimum of 75 subjects, a total of 83 subjects were recruited and divided into three groups, of which, 25 were normal subjects, 32 were diabetics without retinopathy and 26 were mild-to-moderate NPDR diabetics. Only one eye with the highest image quality was selected for each subject, and thus, 42 right eyes and 41 left eyes were analyzed.

### 3.1. Demographic Data

There were 54 (65.1%) female and 29 (34.9%) male subjects. The demographic data are shown in Table 1. The mean age was 52.4 ± 10.3 years (age range of 35 to 73 years) for normal subjects, 58.5 ± 6 years (age range of 47 to 69 years) for diabetics without DR, and 58.4 ± 3.3 years (age range of 52 to 65 years) for mild-to-moderate NPDR diabetics. There was no significant difference in the mean age between the groups. There were more female subjects than male subjects. There was a significant difference in the mean diabetes duration between the mild-to-moderate NPDR group and the diabetic without retinopathy group (t (56) = −5.80, *p* < 0.05). This result suggests subjects with a longer duration of diabetes tend to have a more severe stage of DR. 

**Figure 3:**
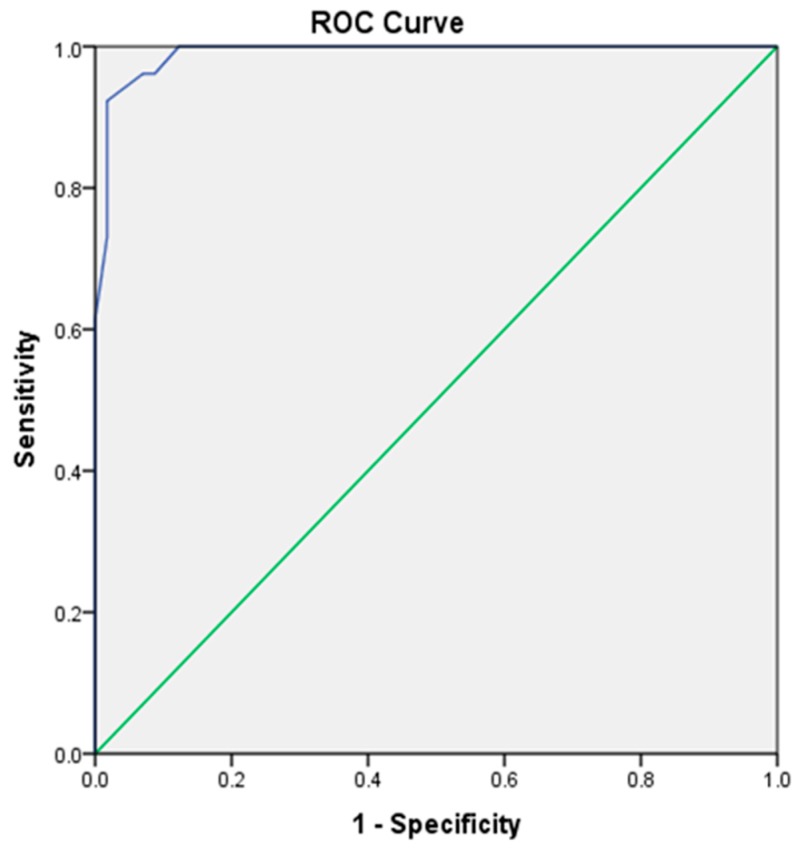
Receiver operating characteristic (ROC) curve of hemoglobin A1c (HbA1c) levels to detect diabetic retinopathy (DR).

Similarly, there was also a significant difference in the mean HbA1c levels between the mild-to-moderate NPDR group and the diabetic without retinopathy group (t (56) = −11.32, *p* < 0.05). Patients with higher HbA1c levels tend to have changes in the retina. A receiver operating characteristic (ROC) curve was plotted to determine the cut-off point of HbA1c to detect DR, as shown in Figure 3. Several choices of HbA1c levels were compared to choose the optimal cut-off point to detect DR, as shown in Table 2. The HbA1c level range of 6.9–7.1 was considered a good cut-off point to detect DR because the sensitivity and specificity exceeded 90%. The area under the curve (AUC) was almost perfect (0.99), which reveals that HbA1c is a good indicator, capable of identifying subjects with and without DR correctly by approximately 99%.

### 3.2. Hyper-Reflective Dots (HRD) Count

The number of HRD was counted manually by two masked examiners. Cohen’s kappa coefficient was used to measure the inter-rater agreement for image grading of the HRD. The intra-grader and inter-grader agreements were evaluated. There was very strong agreement, according to Landis and Koch (1977), for inter-rater and intra-rater agreements, κ = 0.841 (95% CI), *p* < 0.05 and κ = 0.871 (95% CI), *p* < 0.05 respectively.

A one-way ANOVA test was conducted to compare the HRD count between the normal group, the diabetics without retinopathy group, and the mild-to-moderate NPDR group [F (2, 80) = 183.45, *p* < 0.05]. Post hoc comparisons using the Tukey test indicated that the mean HRD count for the normal group (0.48 ± 0.87) was significantly different to the diabetic without retinopathy group (10.25 ± 3.37), and the mean number of HRD for the diabetic without retinopathy was also significantly different to the mild-to-moderate NPRD group (14.96 ± 3.08), as shown in Table 3. This result suggests that subjects with a more severe stage of retinopathy tend to have a higher HRD count. The mean HRD count in the inner retina layer (8.58 ± 6.31) was significantly higher compared to the outer retina layer ((0.25 ± 0.60), t = 12.20, *p* < 0.05).

### 3.3. Correlation between HbA1c Levels and HRD Count 

**Figure 4:**
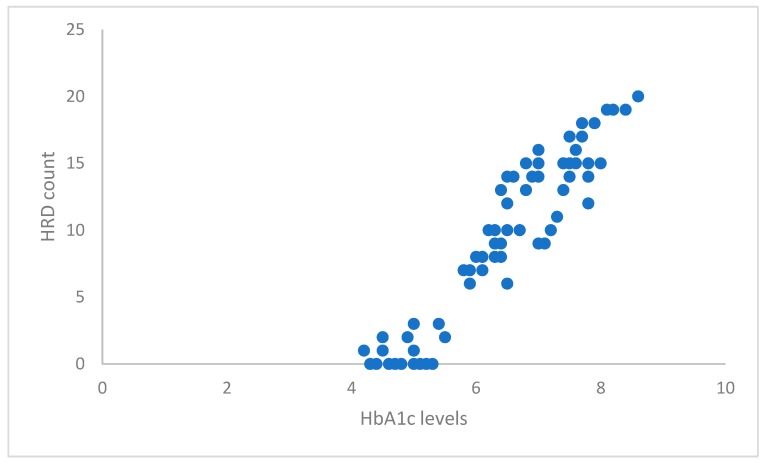
Correlation between HbA1c levels and HRD count.

Figure 4 shows that there is a linear relationship between HbA1c levels and HRD count. A Pearson correlation coefficient was computed to assess the relationship between the HbA1c levels and HRD count, and a strong, positive correlation was found (r = 0.952, n = 83, *p* < 0.05).

### 3.4. HbA1c Cut-Off Level for the Appearance of HRD

**Figure 5:**
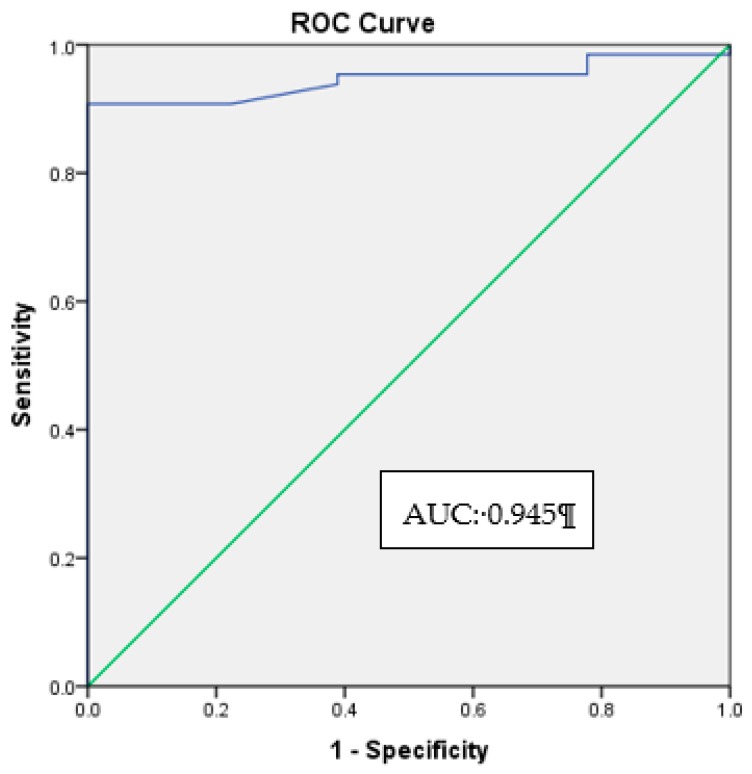
ROC curve and area under the curve (AUC) for the appearance of HRD.

As an initial step towards determining the HbA1c cut-off level for the appearance of HRD, a ROC curve was created using the normal and diabetic patients. ROC curves show the pairing of true-positive and false-positive coordinates across a range of cut-off points that distinguish positive cases from controls. For the ROC curve, the true-positive rate (sensitivity) represents the proportion of patients with HRD that were correctly classified by the HbA1c. The false-positive rate (1–specificity) represents the proportion of patients without HRD incorrectly classified as having HRD. Figure 5 shows the ROC curve and the area under the curve for the appearance of HRD in diabetic patients. The area under the curve (AUC) was 0.945, which indicates that HbA1c is an excellent indicator, capable of identifying subjects with and without HRD correctly by 94.5%. A few HbA1c levels were selected to compare and choose the cut-off point with the highest sensitivity and specificity as shown in Table 4. The HbA1c level of 5.4% was chosen as the cut-off point for the appearance of HRD because it has both high sensitivity (89.20%) and specificity (96.60%). 

## 4. Discussion

The present study was conducted to determine the correlation between HbA1c levels and HRD count. The duration of diabetes was shown to be significantly different between the two diabetic groups, namely, the diabetic subjects without retinopathy, and those with mild-to-moderate NPDR. There are many studies that suggest that the duration of diabetes is closely linked to the prevalence of diabetic retinopathy (DR) [19,20,21]. According to the World Health Organization (2016), less than 5% of patients will have retinopathy at diagnosis, while its prevalence can rise up to 40%–50% after ten years. After 20 years of diabetes, most patients with type 1 diabetes, and 60% of patients with type 2 diabetes have some degree of DR. The duration of diabetes is significantly associated with the development and severity of DR, with the odds ratio (OR) ranging from 1.00 to 8.74 in a study conducted by Lim et al. [22]. The OR was 1.00 for less than 3 years, 2.49 for 3 to 6 years, 5.61 for 7 to 11 years, and 8.75 for 12 years or longer. Another study reported OR as high as 8.62 if the duration of DM is more than 15 years [23]. 

In our study, there was a significant difference between the mean value of HbA1c levels in the normal group (4.80 ± 0.34), the diabetic without retinopathy group (6.43 ± 0.41), and the mild-to-moderate NPRD group (7.66 ± 0.41). Our results correlate well with the study of Sabanayagam et al. [24] which mentioned that in subjects with mild-to-moderate NPDR, HbA1c levels in the range of 7.0%–7.9% and ≥8.0% had a nine-fold and 30-fold higher prevalence, respectively, than in subjects with HbA1c ≤ 6.9%. In their study, the optimal cut-off points with maximum sensitivity and specificity to detect mild to moderate DR were 6.6% and 7.0%, and the AUCs were 0.899 and 0.904, respectively. The two cut-off points of sensitivity and specificity were in agreement with other studies which recommended the HbA1c cut-off points of ≥6.5% and 7.0%, respectively [25,26]. However, another two studies showed different cut-off points, which were in the range of 6.0% to 7.0% [27,28]. For our study, the range of cut-off points was 6.9% to 7.1%. The possible reasons for the variability in optimal cut-off points could be due the different assay methods, such as ion exchange chromatography, boronate affinity and immunoassays [29]. We used ion exchange chromatography as the assay, as with this method chromograms can be inspected for hemoglobin variants, and it is very precise. Boronate affinity has less interference from hemoglobinopathies, but it measures glycated α and β chains which may affect readings. Immunoassays are relatively easy to implement under many different formats, but are affected by hemoglobinopathies [30]. Gallagher et al. [31] documented several other factors that induce variable HbA1c levels such as erythropoiesis, altered hemoglobins, glycation (alcoholism, aspirin, vitamin C and E, genetic determinants), and erythrocyte destruction (drug-induced).

Our present study reported the presence of HRD and their location (inner retina or outer retina layer) in normal subjects, diabetic subjects without retinopathy, and subjects with mild-to-moderate NPDR on linear B-scans (SD-OCT), and compared these to en face fundus photo to exclude hard exudates, microaneurysm, and blood vessels. In our study, the presence and number of HRD increased in diabetic subjects without retinopathy, and especially in diabetic subjects with clinical signs of DR (mild-to-moderate NPDR), when compared with normal subjects. HRD were first described in a study conducted in 2012 [32]. They were described as small, punctiform hyper-reflective spots that are scattered throughout all retina layers (mostly in the outer retina) in late stage age-related macular degeneration (AMD). The authors described HRD as activated microglial cells, because the number of HRD was reduced quickly after anti-inflammatory injection treatment, providing optimal improvement in the visual acuity of subjects. Thus, the amount of baseline HRD was suggested to be a predictive factor for therapy outcome, and to demarcate the severity of the disease. A previous study reported the increase in HRD in diabetic subjects versus normal subjects and diabetic subjects without any clinical retinopathy [11]. They suggested that HRD may represent aggregates of activated microglial cells that have migrated from more inner retina layers to more outer retina layers depending on the progression of the disease. Another study proposed that HRD in the outer retina in diabetic macular edema were closely associated with the disrupted external limiting membrane and inner or outer segment lines, suggesting that they originated from degenerated photoreceptors, or from macrophages engulfing them [33]. Therefore, our study which mostly consists of subjects with early diabetic changes, may not have a significant presence of HRD in the outer retina layer because the external limiting membrane and inner or outer segment lines may still remain intact. 

We also report that HRD were significantly more numerous in the inner retina (IR) layer of diabetics, and almost absent in the outer retina (OR) layer of subjects in the normal group. This is because the resting retinal microglia is physiologically located in the IR layers, and the activation process begins in, and expands from the IR layers before migrating toward the OR layer [34]. In our study, it is likely that HRD are activated microglial cells because of the location in the inner retina (where the resident microglia are present), lack of back shadowing, small size, and the absence of vessels or any other lesion in the en face fundus photos. This is also documented in a study which showed a similar HRD presence [11].

An almost perfect correlation (r = 0.952, *p* < 0.05) between HbA1c levels and number of HRD was achieved in our study. Overall, the increase in the number of HRD correlated with the increase in HbA1c levels. This was supported by a study which showed that the baseline HRD amount correlated positively with HbA1c values, and indicated the severity of disease [35]. Two studies were completed to interpret HRD as activated microglial cells which were caused by chronic inflammation in the retina [33,34]. This chronic inflammation is more likely caused by hyperglycemia or poor control of blood glucose levels [36]. This low-level inflammation may be tolerated by diabetic patients for years without causing any damage, but the accumulating alteration may ultimately break the tolerance and worsen DR. Two studies showed that insulin resistance and type 2 diabetes are associated with higher levels of C-reactive protein (CRP), interleukin-6 (IL-6), and tumour necrosis factor-α (TNF- α), which are markers of subclinical systemic inflammation [37,38]. Thus, we used HbA1c as an indicator of retinal inflammation (hyperglycemia) and tested the correlation with presence of HRD (activated microglial cells). According to our results, we can conclude that hyperglycemia could be the reason behind the activation of microglial cells and the increase in the appearance of HRD.

## 5. Conclusions

Our study has shown that HRD count increases with the severity of HbA1c levels. There was a positive linear correlation between HbA1c levels and the presence of HRD. This indicates that hyperglycemia may activate microglial cells in inner retina, thus causing a chronic level of retinal inflammation. This study has also determined the HbA1c cut-off level for the appearance of HRD in diabetics. The AUC from the ROC curve was 0.945, which indicated that HbA1c is an excellent indicator, capable of identifying diabetic subjects with and without HRD correctly by approximately 94.5%. The optimal cut-off point for the appearance of HRD is the HbA1c level of 5.4%. This suggests that when a diabetic patient has a HbA1c level higher than 5.4%, the chances of the patient having an appearance of HRD is extremely high. Thus, HbA1c is correlated with the appearance of HRD and, is therefore, an excellent predictor for the appearance of HRD in diabetics, which could be caused by the activation of microglial cells. 

## Figures and Tables

**Table 1:** Demographic data of subjects (n = 83).

Characteristics	Frequency (%)	Mean ± SD Confidence Interval	Normal (n = 25)	Diabetic without Retinopathy (n = 32)	Diabetic with Mild to Moderate NPDR (n = 26)
Age (years)		Mean ± SD (Range)	52.40 ± 10.30	58.50 ± 6.04	58.38 ± 3.35
		95% Confidence Interval	48.2–56.7	56.3–60.7	57.0–59.7
Gender					
Male	29 (34.9)		11	11	7
Female	54 (65.1)		14	21	19
Race/ethnicity					
Malay	54 (65.1)		16	23	15
Chinese	12 (14.5)		6	3	3
Indian	17(20.5)		3	6	8
Other	0		0	0	0
Diabetes duration (years)		Mean ± SD (Range)		6.6 ± 1.6	9.0 ± 1.6
		95% Confidence Interval		6.0–7.1	8.4–9.6
HbA1c level		Mean ± SD (Range)	4.80 ± 0.34	6.43 ± 0.41	7.66 ± 0.41
		95% Confidence Interval		6.3–6.6	7.5–7.8

**Table 2:** Sensitivity and specificity of each HbA1c level to detect DR.

HbA1c Level	Sensitivity (%)	Specificity (%)
6.80	100.00	87.70
6.90	96.20	91.20
7.00	96.20	93.00
7.10	92.30	98.20
7.20	88.50	98.20

**Table 3:** Comparison of number of HRD.

Groups	HRD Count (Mean ± SD)			T-Test (Total IR vs. OR)	
	Inner Retina (IR)	Outer Retina (OR)	Total	t-Value	*p*-Value
Normal	0.48 ± 0.87	0	0.48 ± 0.87	12.20	<0.05
Diabetic without retinopathy	10.16 ± 3.30	0.09 ± 0.39	10.25 ± 3.37		
Mild-to-moderate NPDR	14.42 ± 3.50	0.54 ± 0.90	14.96 ± 3.08		

**Table 4:** Sensitivity and specificity of each HbA1c level for the appearance of HRD.

HbA1c Level (%)	Sensitivity (%)	Specificity (%)
4.90	95.40	61.10
5.00	93.80	61.10
5.10	90.80	77.80
5.20	90.80	83.30
5.30	90.80	94.40
5.40	89.20	96.60
5.50	87.10	96.60
